# The gene expression patterns as surrogate indices of pH in the brain

**DOI:** 10.3389/fpsyt.2023.1151480

**Published:** 2023-05-02

**Authors:** Hideo Hagihara, Tomoyuki Murano, Tsuyoshi Miyakawa

**Affiliations:** Division of Systems Medical Science, Center for Medical Science, Fujita Health University, Toyoake, Japan

**Keywords:** postmortem brain, pH, gene expression patterns, meta-analysis, neuropsychiatric disorders, neurodegenerative diseases

## Abstract

Hydrogen ion (H^+^) is one of the most potent intrinsic neuromodulators in the brain in terms of concentration. Changes in H^+^ concentration, expressed as pH, are thought to be associated with various biological processes, such as gene expression, in the brain. Accumulating evidence suggests that decreased brain pH is a common feature of several neuropsychiatric disorders, including schizophrenia, bipolar disorder, autism spectrum disorder, and Alzheimer's disease. However, it remains unclear whether gene expression patterns can be used as surrogates for pH changes in the brain. In this study, we performed meta-analyses using publicly available gene expression datasets to profile the expression patterns of pH-associated genes, whose expression levels were correlated with brain pH, in human patients and mouse models of major central nervous system (CNS) diseases, as well as in mouse cell-type datasets. Comprehensive analysis of 281 human datasets from 11 CNS disorders revealed that gene expression associated with decreased pH was over-represented in disorders including schizophrenia, bipolar disorder, autism spectrum disorders, Alzheimer's disease, Huntington's disease, Parkinson's disease, and brain tumors. Expression patterns of pH-associated genes in mouse models of neurodegenerative disease showed a common time course trend toward lower pH over time. Furthermore, cell type analysis identified astrocytes as the cell type with the most acidity-related gene expression, consistent with previous experimental measurements showing a lower intracellular pH in astrocytes than in neurons. These results suggest that the expression pattern of pH-associated genes may be a surrogate for the state- and trait-related changes in pH in brain cells. Altered expression of pH-associated genes may serve as a novel molecular mechanism for a more complete understanding of the transdiagnostic pathophysiology of neuropsychiatric and neurodegenerative disorders.

## Introduction

As hydrogen ions (H^+^) can act as neuromodulators, brain acid-base homeostasis, which is differentially regulated in different cell types, is considered essential for proper function in each cell type (1, 2). Alterations in brain pH have been implicated in several diseases. In particular, accumulating evidence from *in vivo* and *in vitro* studies has suggested decreased brain pH in schizophrenia (SZ), bipolar disorder (BD), autism spectrum disorder (ASD), and Alzheimer's disease (AD) (3–11), as well as in brain tumors (BT) (12, 13). There are also studies reporting a significant decrease or trend toward a decrease in brain or spinal cord pH in Parkinson's disease (PD), amyotrophic lateral sclerosis (ALS), and alcoholism (ALC) (14–17).

Tissue pH has been considered as an indicator of the quality of preserved tissues and a variable that is confounded by some antemortem and postmortem conditions, especially agonal experiences (18–21). Therefore, considerable efforts have been made to match tissue pH between cases and controls to be compared and to control for the effect of pH on molecular changes in postmortem studies. Studies with unbiased tissue collection regarding tissue pH are limited (e.g., studies that did not clearly state in the paper that the pH of the tissue samples used were matched between cases and controls). However, it is controversial whether changes in brain pH reflect a primary feature of the diseases or are the result of confounding factors. While several pre- and postmortem factors have been suggested to affect brain pH (such as sex, agonal state, age at death, and postmortem interval), the results are inconsistent; some studies have shown slightly higher brain pH in normal healthy males than females (20), while others have shown no sex differences in both patients with AD and control subjects (22). Prolonged agonal states, such as extended periods of respiratory arrest and multi-organ failure and coma, have been shown to correlate with lower brain pH (19–21, 23, 24), but no significant effect of agonal state on brain pH has also been reported (22, 25). Decreased brain pH has been observed in patients with BD compared to healthy controls, where most of the subjects analyzed in the study experienced relatively rapid death, suggesting that decreased brain pH may be directly related to BD disease (26). Regarding the effect of age on brain pH, some studies showed no significant correlation between brain pH and age in the range of 0 to 92 years (20), 23 to 96 years (24), and 41 to 102 years (22), while others showed a negative correlation in postmortem (17 to 92 years) (19) and MRS (21 to 84 years) (27) studies. No significant correlation between brain pH and postmortem interval has been observed in the groups of control subjects (19, 20, 22, 25) and the combined group of patients with SZ and controls (24), while a modest but significant correlation has been found in patients with AD (22). Brain pH has been shown to correlate with RNA integrity, and therefore pH has been used as an indicator of the quality of preserved tissue (18, 19, 24, 25, 28, 29). However, it has also been suggested that a higher pH does not necessarily guarantee intact RNA (30).

It is technically difficult to exclude the potential effects of such variables on brain pH in human studies, especially those using postmortem brain samples. Therefore, animal models that are exempt from such confounding factors may help to confirm whether changes in brain pH are involved in the pathophysiology of neuropsychiatric and neurodegenerative disorders. Our recent large-scale analysis showed that decreased brain pH, which correlates with increased levels of metabolic acid lactate, is a common feature in various animal models of neuropsychiatric and neurodegenerative disorders, supporting the idea that the phenomenon reflects an underlying pathophysiology of the disorders rather than a mere artifact (31). Given that decreased brain pH is a pathological trait of certain psychiatric disorders, controlling for the effect of pH on molecular changes may have inadvertently obscured the specific pathological signatures potentially associated with pH changes, such as neuronal hyperexcitation and inflammation, both of which have been implicated in the etiology of neuropsychiatric disorders. pH-dependent changes in gene expression are of concern when attempting to elucidate the molecular basis of the disorders.

Regarding the mechanisms of decreased brain pH, studies in humans and animals have suggested that decreased brain pH is associated with increased lactate concentrations (3, 4, 31, 32). It is hypothesized that increased brain lactate concentrations in SZ and BD occur as a result of mitochondrial dysfunction, with a shift away from the tricarboxylic acid cycle and oxidative phosphorylation toward increased glycolysis for energy production (5–7, 33, 34). In addition, prolonged neural activity has been suggested to induce intracellular acidification due to increased production of metabolic acids such as lactate and CO_2_ (2). The uptake of the neurotransmitter glutamate following neuronal activation also stimulates glycolysis in astrocytes, resulting in increased lactate production (35). Mitochondrial dysfunction and neuronal hyperexcitation in specific brain regions have been implicated in the etiology of SZ (36–40), BD (36, 41–45), AD (46–49), and other neuropsychiatric disorders. Although the functional significance of pH changes in neuropsychiatric disorders is unclear, brain acidosis has been suggested to affect the regulation of neurotransmitter systems; *in vitro* studies have shown that acidosis inhibits dopamine reuptake (50) and impairs excitatory synaptic transmission at cortical GABAergic neurons (51), which may lead to brain dysfunction associated with neuropsychiatric disorders. Clinically, low pH has been shown to be correlated with lower IQ (52) and greater AD severity (53).

Genome-wide gene expression analysis of postmortem tissues has provided valuable information on the molecular mechanisms of brain disorders. To date, many hypothesis-based (54–57) and hypothesis-free (58, 59) characterizations of transcriptomic signatures have proposed potential molecular mechanisms of neuropsychiatric and neurodegenerative disorders. For example, based on the hypothesis that neural hyperexcitation and pseudoimmaturity in certain brain regions are involved in the pathogenesis of neuropsychiatric disorders, we previously performed an analysis of transcriptomic profiles of neuropsychiatric disorders (56). Specifically, we first identified neural hyperexcitation-related genes whose expression level was increased or decreased in the hippocampus of rats with pilocarpine-induced seizures compared to saline-treated rats, and maturation-related genes whose expression level decreased (referred to as immaturity marker genes) or increased (referred to as maturity marker genes) with maturation in the human fatal hippocampus. We then defined two gene expression patterns: hyperexcitation-induced increase in immaturity marker genes (hiI genes) and hyperexcitation-induced decrease in maturity marker genes (hiM genes). The hiI/hiM genes were used to evaluate the degree of similarity of expression changes between the two sets of genes and genes differentially expressed in neuropsychiatric disorders (patients compared to unaffected controls) (56). In the analysis, we found that the patterns of expression changes in hiI and hiM genes were highly similar to those in SZ, AD, and ASD, suggesting that transcriptomic pseudoimmaturity induced by neural hyperexcitation is shared by several neuropsychiatric disorders (56). Then, in the present study, using the same methodological approach developed in our previous study (56), we investigated whether and to what extent the expression changes in pH-associated genes (i.e., pH-upregulated genes and pH-downregulated genes) overlap with those in neuropsychiatric disorders.

Although the effect of pH changes has also been demonstrated at the transcriptional level in the brain (24, 60, 61), whether gene expression patterns can be surrogates for brain pH changes remains unknown. In the present study, we developed an “acid-gene index” and an “alkaline-gene index” based on the expression patterns of previously identified pH-downregulated genes and pH-upregulated genes, whose expression levels were negatively or positively correlated with pH values, in the human brain. Using these indices, we comprehensively evaluated whether, and to what extent, the expression patterns of pH-associated genes overlap and are similar to those of genes differentially expressed in major human central nervous system (CNS) disorders and in mouse models of these disorders. We also evaluated the overlap and similarity of expression of these genes with cell type-dependent expression in mice. Despite the variety of sample types used, we were able to identify some common and distinct patterns of gene expression in the human disease and mouse cell type datasets. This was largely due to the use of a bioinformatics method, the Running Fisher algorithm (62), which employs a normalized, fold-change-based ranking approach and thus allows measurement of comparability across data from different studies, species, and analysis platforms. Although neuropsychiatric and neurodegenerative disorders clinically fall into different diagnostic categories, some phenotypic and biological features, such as genetic mutations, molecular alterations, and changes in brain activity (63–67), as well as changes in brain pH, have been suggested to be shared among them. This study suggests that gene expression signatures associated with changes in brain pH can be observed transdiagnostically in several neuropsychiatric and neurodegenerative disorders, especially those that show decreased brain pH.

## Materials and methods

### pH-associated genes

Cross-laboratory analysis of genome-wide expression data from normal human postmortem cortex provides a source of information on pH-associated gene expression (60). The “normal human” was defined as follows for the datasets of Stanley Kato and Stanley Chen, which were used to identify pH-associated genes in the study by Mistry and Pavlidis (60): “Diagnoses of unaffected controls were based on structured interviews by a senior psychiatrist with family member(s) to rule out Axis I diagnoses” (https://www.stanleyresearch.org/brain-research/). The meta-analysis identified genes whose expression levels were robustly correlated with tissue pH from four studies (60). In this study, we used genes with a meta-analysis *Q*-value < 0.001 [39 pH-upregulated genes whose expression levels were positively correlated with pH values and 268 pH-downregulated genes whose expression levels were negatively correlated with pH values; see Supplementary Table 6 in Mistry and Pavlidis (60)]. The subset of genes (gene name and *S*-value, which was treated as a fold change value in the current study) was imported into the bioinformatics tool BaseSpace (62) according to the manufacturer's instructions.

### Pathway enrichment analysis

Pathways/biogroups enriched in the genes of interest were determined by a combination of rank-based enrichment statistics and biomedical ontologies using BaseSpace (62). Pathways/biogroups from GO and canonical pathways from Broad MSigDB were included in this analysis.

### Gene expression patterns in the cortex of individual subjects

We used two datasets from the Stanley Medical Research Institute online genomics database (https://www.stanleygenomics.org/) and six datasets from the Gene Expression Omnibus (GEO) repository at the National Center for Biotechnology Information (NCBI) archives (https://www.ncbi.nlm.nih.gov/geo/), where raw microarray data and tissue pH of individual subjects were publicly available: Stanley Kato (normal = 34, SZ = 35, BD = 32) (36), Stanley Chen (normal = 13, BD = 14), GSE2164 (normal = 10) (68), GSE11512 (normal = 13) (69), GSE5392 (normal = 31, BD = 30) (70), GSE161986 (normal = 17, ALC = 18) (71), GSE21935 (normal = 19, SZ = 23) (72), and GSE84422 (normal = 15, AD = 41) (73). The inclusion/exclusion criteria of the samples in the disease studies used in this analysis are as follows: Patients with SZ and BD included in the studies of Stanley Kato, Stanley Chen, GSE5392, and GSE21935 were diagnosed according to the Diagnostic and Statistical Manual of Mental Disorders, 3rd or 4th edition, and had no history of significant focal neurological signs premortem. In the study of GSE161986, samples with a history of infectious disease, circumstances surrounding death, substantial brain damage, and postmortem interval >48 h were excluded. In the study of GSE84422, each case was assessed according to a Braak AD-staging score for progression of neurofibrillary neuropathology. All cases were assessed and diagnosed according to relevant clinical methods. It is highly unlikely that these patients had comorbid neurological diseases. We calculated the relative expression level of each gene for each subject within the study in which the subject was enrolled. Using the expression values, we calculated the fold change of each gene in a sample (test sample) compared to the mean expression value of the remaining samples within a given study. This was repeated until all samples were selected once as the test sample. For example, in the Stanley Kato dataset (101 samples in total), sample #1 and the remaining 100 samples (#2–#101) were treated as test and control samples, respectively. The mean expression value was calculated for each gene in the control samples. Then, the fold change of each gene of the test sample was calculated by comparing the expression value of the test sample with the mean expression value of the control samples. These procedures constituted the first round and were repeated for 101 rounds so that all 101 samples could be selected once as the test sample. Genes with an absolute fold change of >1.2 were considered as differentially expressed genes (DEGs). We imported the DEG lists of 345 subjects into BaseSpace (62) to obtain the acid-gene index and alkaline-gene index for each subject (see the section “Acid-gene index and alkaline-gene index” below). We then examined the correlation of each of the obtained indices with the subject's brain pH.

### Genome-wide expression datasets of human CNS disorders

All gene expression datasets used in this study have been previously published and are publicly available and have been included in the BaseSpace database (Supplementary Table 1). The database contains more than 128,000 RNA expression datasets (lists of DEGs in the test group compared to the corresponding control group) obtained from the tissues and cells of humans, mice, and other species. We comprehensively queried human (*Homo sapiens*) RNA expression datasets from the BaseSpace database using disease names as keywords (e.g., “schizophrenia”, “bipolar disorder”, “Alzheimer's disease”, and “brain tumor”). Datasets with an extremely small number of DEGs were excluded. Datasets from *in vitro* culture studies were also excluded for direct comparison with tissue pH-associated genes. In addition, we obtained the recent hippocampal expression data of schizophrenia, bipolar disorder, and major depressive disorder (MDD) from http://research.libd.org/dg_hippo_paper/data.html (74), imported the lists of DEGs with *P* < 0.05 (patients vs. controls) into BaseSpace, and used them for subsequent analyses.

### Time-course expression datasets from neurodegenerative animal models

We searched the time-course expression datasets of neurodegenerative animal models in the BaseSpace database. The time-course expression datasets consisted of the microarray data analyzed between mutant and wild-type mice at different ages from young to older adult. We used the following datasets in this study: transgenic mice with heterozygous and homozygous human amyloid precursor protein (APP) and presenilin1 (PSEN1) mutations, transgenic mice with heterozygous microtubule-associated protein tau (MAPT) mutation (cortex and hippocampus in 8-, 16-, 32-, and 72-week-old mice; GSE64398) as AD models; transgenic mice with human superoxide dismutase (SOD1) mutation (spinal cord in 28-, 42-, 56-, 70-, 98-, 112-, and 126-day-old mice; GSE18597, GSE46298) as an ALS model; and transgenic mice expressing mutant huntingtin (HTT) (striatum in 2-, 7-, and 11-month-old mice; GSE64386) as a Huntington's disease (HD) model.

### Hyperexcitation-induced immaturity and maturity-related genes (hiI/hiM genes)

The hiI/hiM genes were obtained from our previous study (56). To examine the developmental changes in gene expression in the human hippocampus, we compared a microarray data from 8–9 weeks fetuses with a data from 10–12, 13–15, 16–18, 19–23, and 24–38 weeks fetuses (GSE25219) (56). In this analysis, immaturity- and maturity-related genes were considered as genes whose expression levels were higher or lower in younger compared to older subjects, respectively. Within the entire fetal hippocampal datasets, the largest overlap for changes in gene expression after pilocarpine injection (pilocarpine-treated rats compared to control rats) was for the comparison between 8–9 weeks fetuses and 19–23 weeks fetuses [Supplementary Figure 1b of Murano et al. (56)]. Genes that were upregulated by neuronal hyperexcitation in the hippocampus of pilocarpine-induced seizure model rats (pilocarpine-treated rats compared with control rats) and upregulated in the human hippocampus of younger individuals (8–9 weeks fetuses compared with 19–23 weeks fetuses) were defined as hiI genes, and genes with expression changes in the opposite direction by these conditions were defined as hiM genes.

### Evaluation of similarity of gene expression patterns

We compared the expression patterns of pH-upregulated and pH-downregulated genes with those of the queried datasets using the Running Fisher algorithm in BaseSpace (62). This algorithm is a non-parametric rank-based statistical method that takes into account rank information based on the absolute value of the fold change and the direction of expression changes within each gene set (62). The greater the similarity of gene expression patterns between the two conditions, the lower the resulting overlap *P*-value (55–57, 75–78). For the cell type analysis data, the fold enrichment of the expression level in one cell type over the other cell types was treated as the fold change of the genes.

### Acid-gene index and alkaline-gene index

**Figure 1:**
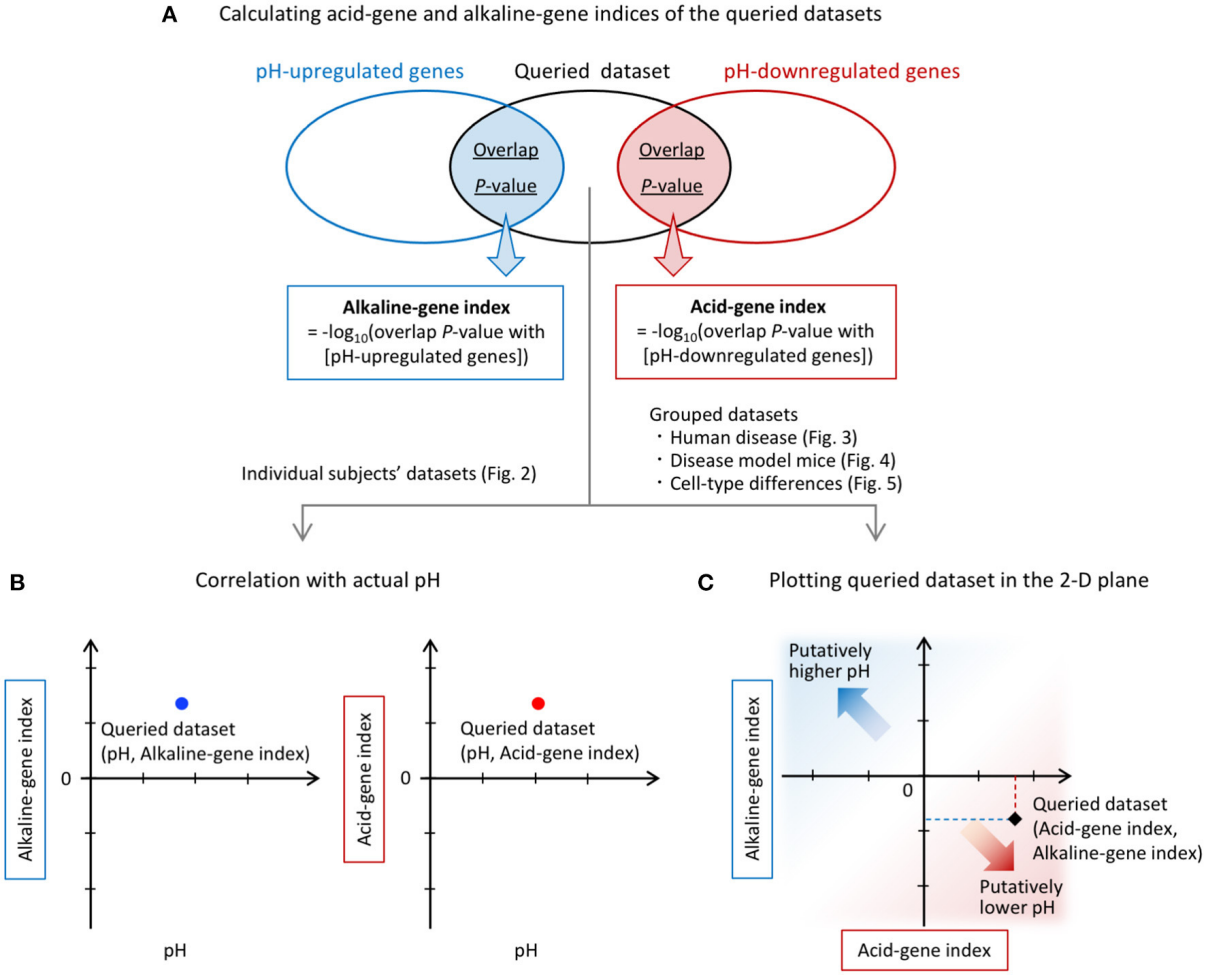
Overview of the analysis. **(A)** Calculation of acid-gene and alkaline-gene indices of the queried datasets. pH-up/downregulated genes obtained from a previous study (60) were compared with each of the DEGs in the queried datasets. The overlap *P*-values were calculated for each comparison using the Running Fisher algorithm. The overlap *P*-values were transformed to alkaline-gene and acid-gene indices, respectively. **(B)** Individual subject datasets were used for correlation analysis with tissue pH. **(C)** The alkaline-gene and acid-gene indices were plotted in the two-dimensional (2-D) plane for each dataset in the analysis of grouped datasets.

The calculated overlap *P*-values were transformed to obtain similarity indices, denoted by the acid-gene index and alkaline-gene index, according to the following formulas (Figure 1A):

Acid-gene index = –log_10_ (overlap *P*-value with [pH-downregulated genes])

Alkaline-gene index = –log_10_ (overlap *P*-value with [pH-upregulated genes])

In the analysis of individual human subject datasets, the resulting index values were used to examine the correlations with tissue pH (Figure 1B). In the analysis of grouped datasets, the acid-gene and alkaline-gene indices were plotted in the two-dimensional plane to show the degree of overlap between the queried datasets and the pH-up/downregulated genes (Figure 1C). In this plot, a higher value of the acid-gene index (x-axis) indicates increased expression of pH-downregulated genes, and a lower value of the alkaline-gene index (y-axis) indicates decreased expression of pH-upregulated genes, simulating lower pH in the datasets of interest (Figure 1C).

## Results

### pH-upregulated and -downregulated genes exhibit different biological properties

Pathway enrichment analysis using BaseSpace revealed that the pH-upregulated genes were highly associated with terms related to the synapse (e.g., “chemical synaptic transmission” and “synaptic signaling”) and channel activity (e.g., “regulation of cation channel activity” and “negative regulation of cation channel activity”) (Table 1). The pH-downregulated genes were associated with terms related to cell/tissue development (e.g., “forebrain development” and “gliogenesis”) and extracellular matrix (e.g., “extracellular matrix” and “collagen-containing extracellular matrix”). These results suggest that the increase and decrease of pH may be involved in different aspects of biological processes in the brain.

**Table 1:** Pathway enrichment analysis of pH-up/downregulated genes.

**Biogroup name**	**Source**	**Common genes**	***P*-value**
pH-upregulated genes			
Chemical synaptic transmission	GO	10	9.90E-14
Synaptic signaling	GO	10	1.20E-13
REACTOME_NEURONAL_SYSTEM	Broad MSigDB–Canonical Pathways	8	9.30E-12
REACTOME_TRANSMISSION_ACROSS_ CHEMICAL_SYNAPSES	Broad MSigDB–Canonical Pathways	7	3.10E-11
Neuronal cell body	GO	7	2.10E-08
REACTOME_G_ALPHA_I_SIGNALING_ EVENTS	Broad MSigDB–Canonical Pathways	5	1.30E-07
Regulation of heart contraction	GO	5	1.80E-07
Regulation of cation channel activity	GO	4	2.40E-07
REACTOME_TRANSMEMBRANE_TRANSPORT_OF_SMALL_MOLECULES	Broad MSigDB–Canonical Pathways	6	2.50E-07
Negative regulation of cation channel activity	GO	3	3.20E-07
pH-downregulated genes			
Forebrain development	GO	16	1.10E-12
Positive regulation of cellular component movement	GO	17	5.00E-12
Extracellular matrix	GO	17	8.90E-12
Gliogenesis	GO	12	1.60E-11
Transcriptional repressor activity, RNA polymerase II transcription regulatory region sequence-specific DNA binding	GO	13	1.80E-11
Negative regulation of neurogenesis	GO	13	2.00E-11
Positive regulation of cell motility	GO	16	3.50E-11
Collagen-containing extracellular matrix	GO	13	4.80E-11
Regulation of cell morphogenesis	GO	16	6.40E-11
Heart development	GO	16	7.60E-11

The top 10 terms are shown.

### Expression of pH-associated genes correlates with actual pH in the brain

**Figure 2:**
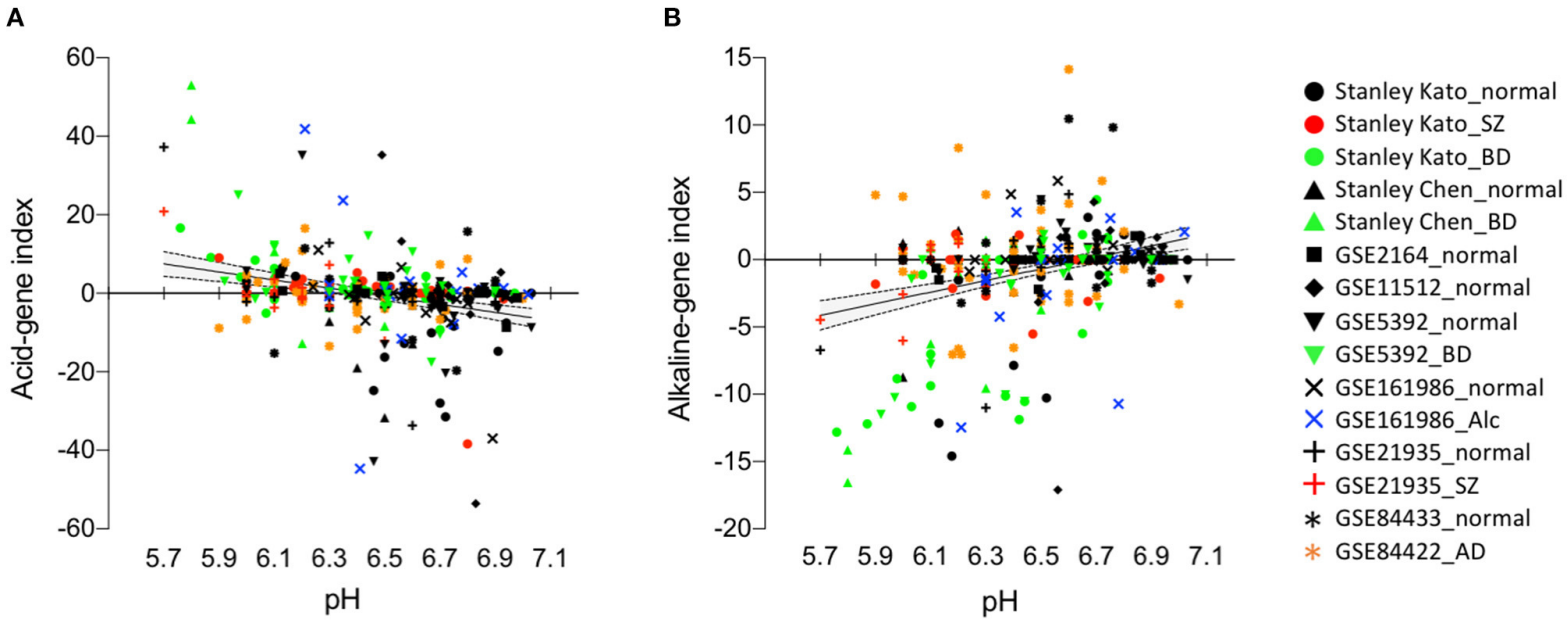
Acid-gene index and alkaline-gene index correlate with actual tissue pH on an individual basis. Scatter plots showing correlations between acid-gene index and tissue pH **(A)** and between alkaline-gene index and tissue pH **(B)**. Each plot represents a single subject. Regression line (solid line) and 95% confidence intervals (dashed line) are shown.

We first evaluate the reliability of our method, i.e., the running Fisher algorithm, to investigate the relationship between the expression patterns of pH-associated genes and the actual pH values (Figures 1A, B). We calculated the acid-gene index and the alkaline-gene index for individual subjects and examined the correlation between actual brain tissue pH and these indices (Figure 2). We analyzed eight cortical datasets consisting of 152 normal subjects, 58 patients with SZ, 76 patients with BD, 18 patients with ALC, and 41 patients with AD in total. Brain tissue pH was significantly decreased in patients with SZ (effect of disease: *P* < 0.0001, effect of batch: *P* < 0.0001, interaction: *P* = 0.046, two-way ANOVA), BD (effect of disease: *P* = 0.0035, effect of batch: *P* < 0.0001, interaction: *P* = 0.82, two-way ANOVA), and AD (*P* = 0.023, unpaired *t*-test), but not patients with ALC (*P* = 0.74, unpaired *t*-test), compared with normal controls. The acid-gene index and the alkaline-gene index were significantly negatively (*r* = −0.28, *P* = 1.60 × 10^−7^) and positively (*r* = 0.33, *P* = 2.78 × 10^−10^) correlated, respectively, with the tissue pH on an individual basis (Figure 2), indicating that the higher the acid-gene index and the lower the alkaline-gene index, the lower the tissue pH, and vice versa. These results confirm the intrinsic relationship between pH-associated gene expression in the brain and the actual tissue pH, and suggest that analysis of the expression patterns of pH-associated genes can surrogate for changes in tissue pH.

### Expression patterns of pH-associated genes in CNS disorders

We then sought to determine whether or to what extent pH-associated genes are involved in human CNS disorders. After a comprehensive search of human datasets of 11 CNS disorders, we found 281 datasets in the BaseSpace database, including 21 datasets of SZ, 16 of BD, 9 of ASD, 18 of MDD, 56 of AD, 23 of HD, 12 of PD, 17 of ALC, 9 of ALS, 11 of multiple sclerosis (MS), and 89 of BT (Supplementary Table 1).

**Figure 3:**
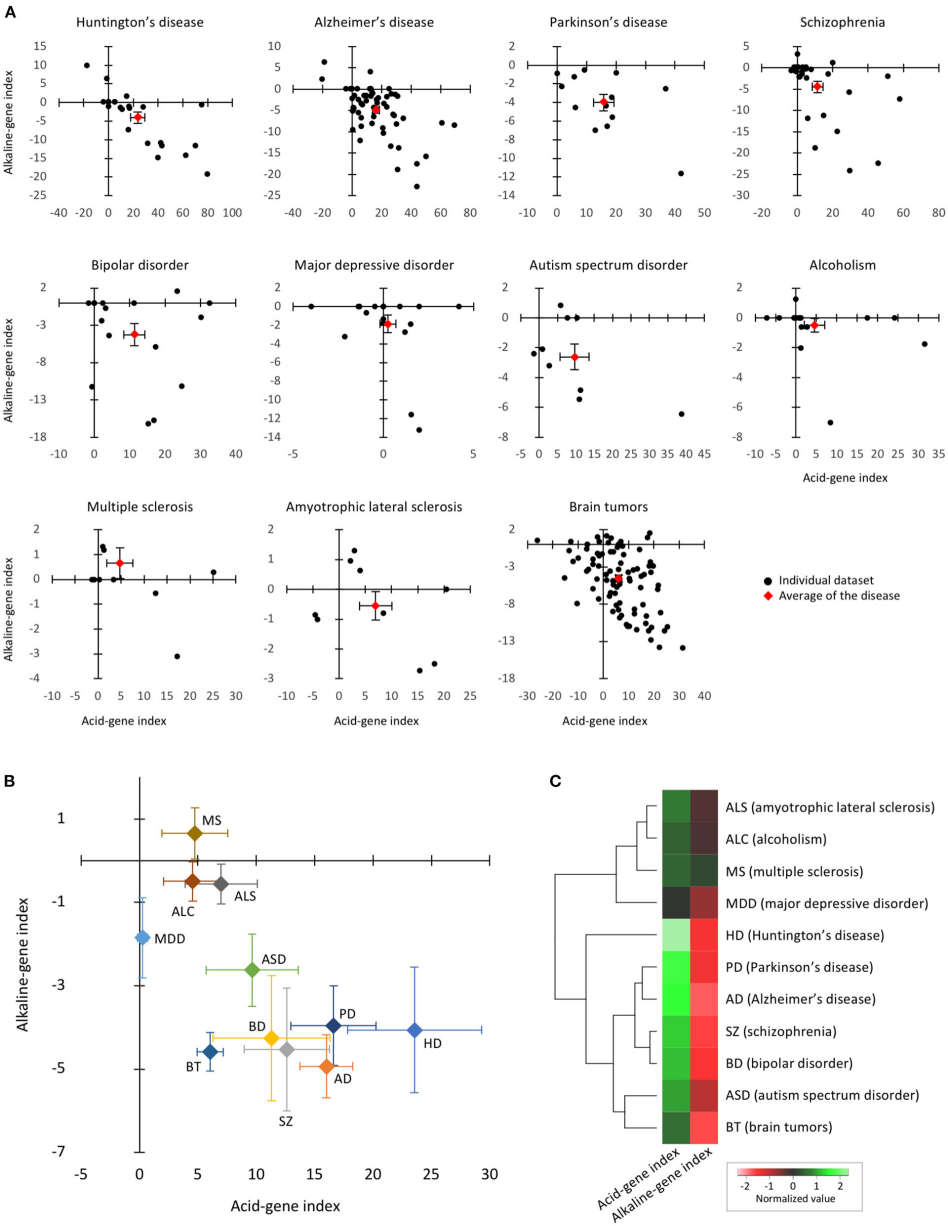
Expression patterns of pH-associated genes in the brain of neuropsychiatric and neurodegenerative disorders. **(A)** The two-dimensional plot results of individual datasets within each disease. Black plots indicate individual datasets and red plots indicate the mean of the datasets in the disease. Error bars are standard errors of the mean. **(B)** The two-dimensional plots of 11 diseases based on the means of the acid-gene and alkaline-gene indices within each disease. Error bars are standard errors of the mean. **(C)** Hierarchical clustering of 11 diseases based on acid-gene and alkaline-gene indices.

The acid- and alkaline-gene indices of each dataset are shown in Figure 3A. The acid-gene and alkaline-gene indices obtained from each dataset were averaged within the disease. The overall distribution patterns of the 11 diseases are shown in Figure 3B. The results showed that the 11 diseases exhibited acid-gene and alkaline-gene indices to different degrees. Notably, seven diseases, HD, PD, AD, SZ, BD, BT, and ASD, had higher acid-gene indices and lower alkaline-gene indices, with a stronger trend in the neurodegenerative diseases HD, PD, and AD. The other four diseases, ALS, ALC, MS, and MDD, showed none to moderate values for both indices. Hierarchical clustering according to the averaged acid-gene and alkaline-gene indices clearly clustered the 11 diseases into the two groups mentioned above (Figure 3C). Given the decreased pH in the diseases, especially in the first group, our results suggest that the expression profiles of pH-associated genes predict experimentally measured tissue pH changes in CNS disorders.

We have previously shown that expression changes of genes related to neuronal hyperexcitation-induced pseudoimmaturity in the brain, which were denoted by hiI/hiM genes, are commonly overrepresented in several neuropsychiatric disorders including SZ, ASD, and AD (56). To investigate whether pH-associated genes and hiI/hiM genes evaluate similar domains of DEGs in these disorders, we examined the overlap between pH-up/downregulated genes and hiI/hiM genes. The results showed that 54 out of 304 pH-associated genes (17.8%) were shared with hiI/hiM genes (Supplementary Figure 1). While the two gene sets showed an overlap *P*-value of 8.5 × 10^−5^, the shared genes were apparently inconsistent in the direction of change between pH-up/downregulated genes and hiI/hiM genes.

### Mapping pH-associated gene expression over time in neurodegenerative disorder models

**Figure 4:**
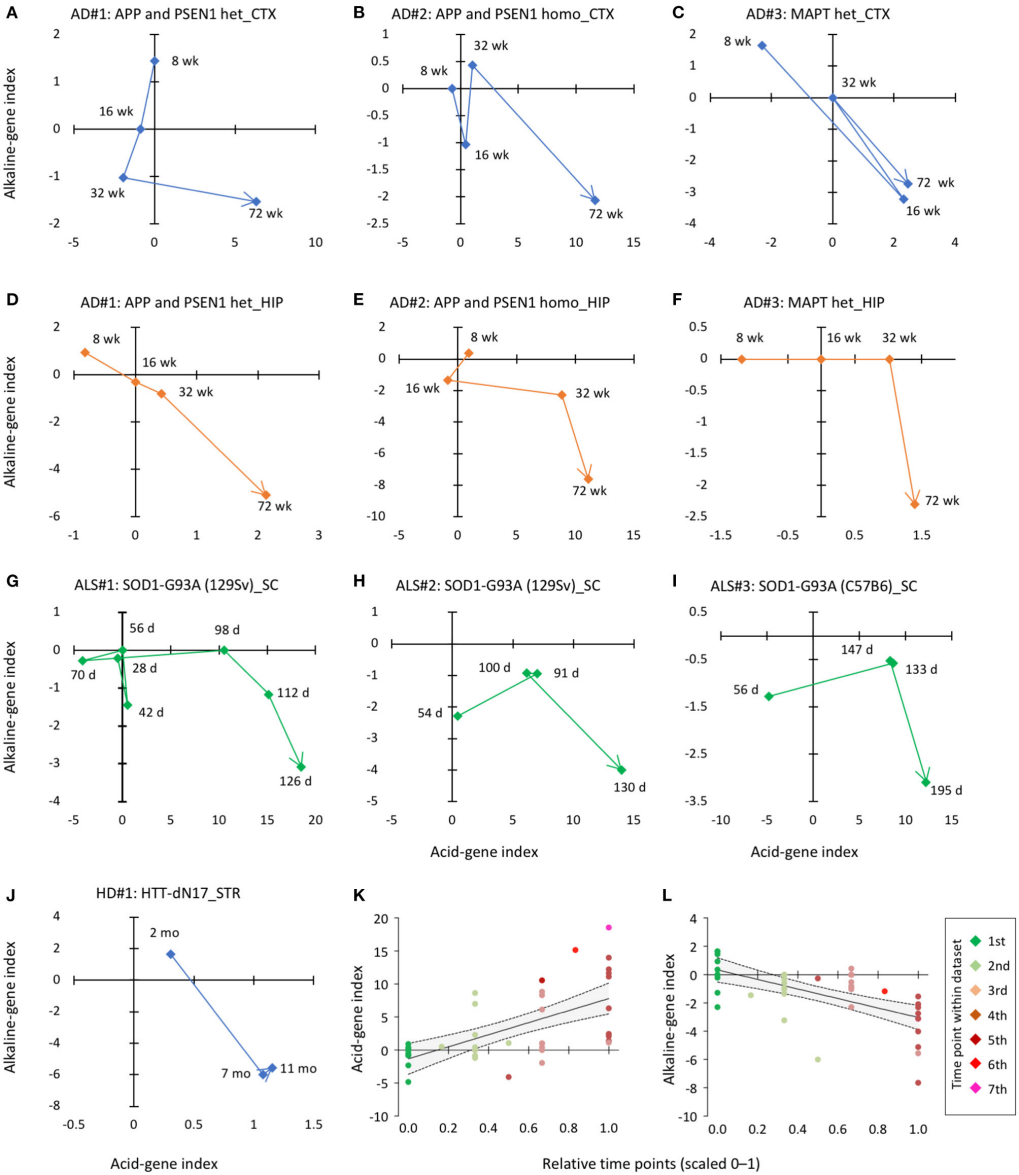
Time-dependent changes in acid-gene and alkaline-gene indices in mouse models of neurodegenerative disorders. **(A–F)** Pattern of changes in acid-gene and alkaline-gene indices in the cortex [CTX; **(A–C)**] and hippocampus [HIP; **(D–F)**] of AD mouse models with heterozygous mutation in APP and PSEN1 **(A, D)**, homozygous mutation in APP and PSEN1 **(B, E)**, and heterozygous mutation in MAPT **(C, F)** (GSE64389). **(G–I)** Patterns of changes in acid-gene and alkaline-gene indices in the spinal cord (SC) of an ALS mouse model with the SOD1 (G93A) mutation (GSE18597, GSE46298). **(J)** Patterns of changes in acid-gene and alkaline-gene indices in the striatum (STR) of an HD mouse model with the huntingtin (HTT) mutation (GSE64386). d, days old; wk, weeks old. **(K, L)** Scatter plots showing the correlation between time point and acid-gene index and alkaline-gene index of ten datasets. Time points were normalized within each dataset and expressed on a scale of 0–1. Regression line (solid line) and 95% confidence intervals (dashed line) are shown.

We then determined whether or when pH-associated genes were expressed in CNS tissues of established animal models of neurodegenerative disease. The availability of the time-course data from disease models in public resources allowed us to examine the short- and long-term effects of risk events on the expression of pH-associated genes. We analyzed the time-course expression data from the cortex and hippocampus of three AD models, striatum of one HD model, and the spinal cord of one ALS model. In this analysis, we found a common trend in the time-course pattern: over time, the acid-gene index increased and the alkaline-gene index decreased (Figures 4A–J). Furthermore, integrated analysis of the ten datasets showed a significant positive correlation between time points and acid-gene index (Figure 4K; *r* = 0.61, *P* = 1.65 × 10^−5^) and a significant negative correlation between time points and alkaline-gene index (Figure 4L; *r* = −0.61, *P* = 1.63 × 10^−5^), confirming that the acid-gene index increased and the alkaline-gene index decreased with time.

### pH-associated gene expression pattern predicts astrocytes as the cell type with the lowest pH

Multiple types of neuronal and glial lineage cells comprise CNS tissues, and the intracellular pH has been suggested to be maintained at different levels in certain types of neurons and astrocytes (79, 80). We investigated cell type differences in the expression of pH-associated genes. To this end, we used a dataset that comparatively examined gene expression levels in 17 cell types of the mouse brain and spinal cord (GSE13379) to calculate acid-gene and alkaline-gene indices for each cell type.

**Figure 5:**
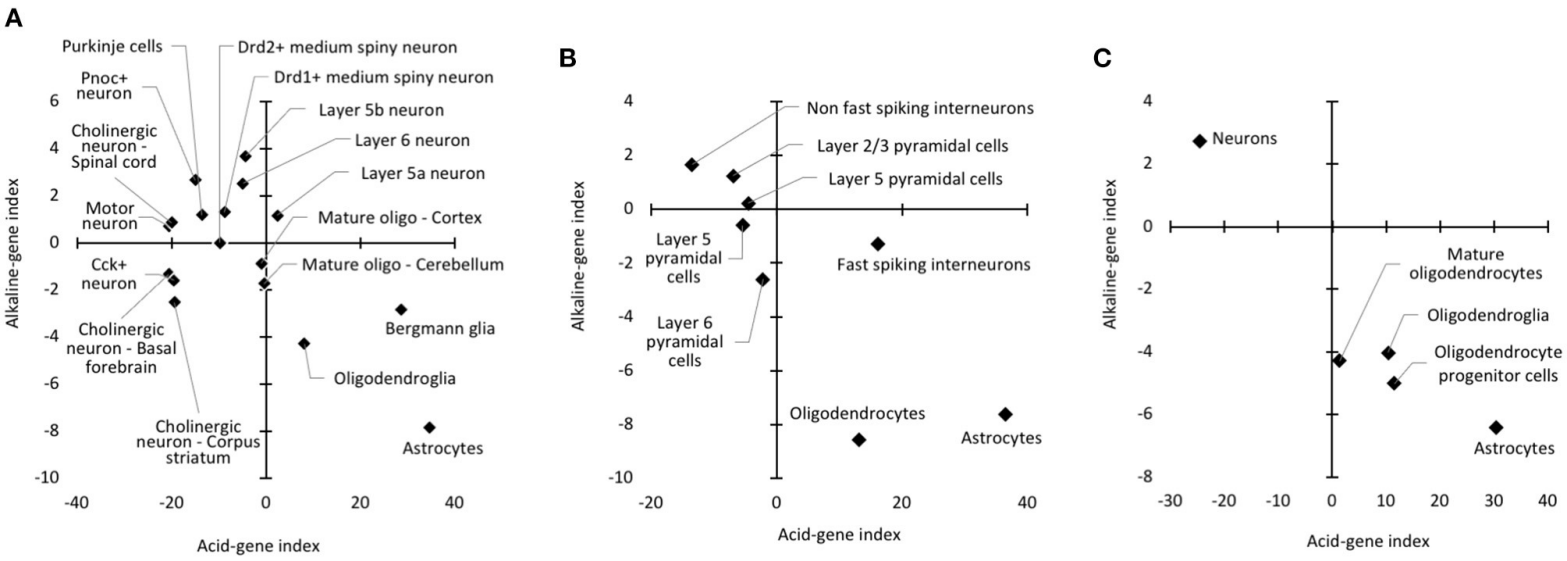
Cell type differences in acid-gene and alkaline-gene indices. Patterns of acid-gene and alkaline-gene indices for each cell type using datasets from GSE13379 **(A)**, GSE21997 **(B)**, and GSE30626 **(C)**.

The results showed that glial cell types tended to have a higher acid-gene index and a lower alkaline-gene index than neuronal cell types. Specifically, astrocytes exhibited the highest acid-gene index and lowest alkaline-gene index among the cell types examined (Figure 5A). Bergmann glia, an astrocyte subtype in the cerebellum, and oligodendrocytes showed similar trends to astrocytes to a lesser extent. In addition, essentially the same results were obtained using other cell type datasets (GSE69340 and GSE30626); glial cell types, especially astrocytes, had a higher acid-gene index and a lower alkaline-gene index than neuronal cell types (Figures 5B, C). These results suggest that astrocytes are a cell type with a lower pH than other neural cell types, as predicted by pH-associated gene expression patterns.

## Discussion

In this study, we provide transcriptomic evidence that the expression of pH-associated genes is overrepresented among the DEGs involved in multiple CNS disorders, supporting the idea of decreased brain pH in these disorders. Furthermore, our results suggest that pH-associated genes are differentially expressed in different cell types. As the pH-associated genes used in this study were identified in neurologically healthy individuals, our results suggest that altered expression associated with pH changes in CNS disorders is an extension of physiologically relevant pH changes.

Pathway enrichment analysis showed that pH-upregulated genes (whose expression levels are positively correlated with pH, with lower expression levels under lower pH conditions) were highly associated with gene ontology terms related to synapse. It has been reported that in mouse hippocampal slice cultures, chronic experimental acidosis reduces spine density and length without affecting dendritic architectures mediated by acid-sensing ion channel 1a (ASIC1a), voltage-independent, H^+^-gated channels (81). This effect was speculated to be due to increased excitotoxicity caused by ASIC1a activation, as acidic treatment induced an ASIC1a-dependent increase in intracellular Ca^2+^ concentration in dendritic spines (82). Furthermore, a recent study showed that intracellular acidification in hippocampal neurons promotes dendritic spine loss and increases stress vulnerability in depression model rats (83). Therefore, extracellular and intracellular acidification may lead to spine impairments at the molecular and cellular levels that may be involved in psychiatric consequences.

In our previous study, we demonstrated that expression changes of genes related to pseudoimmaturity inducible by neuronal hyperexcitation in the brain, which were denoted by hiI/hiM genes, were commonly overrepresented in several neuropsychiatric disorders (56). One might expect that pH-associated genes and hiI/hiM genes would evaluate similar domains of DEGs in these disorders. In fact, only 17.8% of pH-associated genes overlapped with hiI/hiM genes. Furthermore, pathway enrichment analysis showed that hiI genes were highly associated with cell cycle and mitosis (56), which were not found in either pH up or down-regulated genes (Table 1). These results suggest that pH-associated genes and hiI/hiM genes largely evaluated different domains of DEGs in these disorders. However, considering that prolonged neural activity (2) and aging (19, 60) may affect brain pH, we cannot exclude the possibility that these factors may partly contribute to the changes in the expression of pH-associated genes. Further studies are needed to systematically evaluate the relationship between pH changes, neural hyperexcitation, and maturational abnormalities in terms of gene expression patterns and their neurological consequences.

Our analysis of mouse models of neurodegenerative disease showed that the degree of overrepresentation of pH-associated genes increased with age. The severity of symptoms in these mouse models is known to be related to age. In the AD mouse lines studied, both APP/PSEN1 mice and TAU mice showed no apparent amyloid plaques in the hippocampus and cortex at 8 weeks of age and then progressively developed plaques from 4 to 18 months of age (84). Similarly, the SOD1-G93A mouse models of ALS were considered to be in the presymptomatic stage at a mean age of 56 days, began to show signs of symptom exaggeration (impairment in paw grip strength tests and body weight loss) at about 100 days (onset stage), and the disease further progressed thereafter (symptomatic and end stages) (85). These findings suggest that global gene expression changes involving a decrease in brain pH are associated with the progression and severity of the disease-related phenotypes.

The expression patterns of pH-associated genes consistently showed that astrocytes were the cell type with the highest acid-gene expression patterns in the studies using different data sets. As predicted by the gene expression profiles, it has been reported that in mouse hippocampal cultures, the steady-state intracellular pH was lower in astrocytes than in neurons (79, 80). This may be due to differences in the expression or efficacy of Na^+^/H^+^ exchangers (NHEs), Na^+^-driven Cl^−^/${\text{HCO}}_{3}^{-}$ exchangers, Na^+^-${\text{HCO}}_{3}^{-}$ cotransporters (NBCs), and passive Cl^−^/${\text{HCO}}_{3}^{-}$ exchangers between neurons and glial cells (2). Among these genes, we found that SLC4A4 was included in the pH-downregulated genes and enriched in astrocytes in all three cell type datasets examined in this study. SLC4A4, an electrogenic NBC, is predominantly expressed in astrocytes in the mammalian brain and plays a role in regulating intracellular pH in astrocytes (86). Furthermore, we found that pH-associated genes included several genes known to be markers of neuronal and glial cell types; pH-upregulated genes included pan-neuronal markers (SYN2 and GABRA5) and inhibitory interneuron markers (GAD1, NPY, and SST), and pH-downregulated genes included astrocytic markers [AQP4, ALDH1L1, GLUL, SLC1A2 (also known as GLT1), and SLC1A3 (also known as GLAST)]. These results suggest that expression levels of cell type marker genes, as well as genes responsible for regulating intracellular pH, may serve as surrogates for estimating pH changes in the brain. Activation of astrocytes (leading to increased expression of their marker genes) and/or loss of inhibitory interneurons (leading to decreased expression of their marker genes) may potentially contribute to decreased pH-associated gene expression patterns, particularly in the SZ, BD, ASD, HD, and AD [see for example data in references (1, 2, 6–11, 18, 38, 39) in Supplementary Table 1]. It has been suggested that astrocytes produce lactate, a potential major regulator of brain pH, and provide it to neurons as an energy substrate for neuronal activity; this phenomenon is referred to as the astrocyte-neuron lactate shuttle. Furthermore, lactate release has been shown to be stimulated by glutamate uptake in astrocytes following neuronal activation in an *in vitro* study (35). Dysregulation of the excitation–inhibition balance has been implicated in the pathogenesis of several neuropsychiatric disorders, including SZ, BD, and ASD (87, 88). A shift in the balance toward excitation would result in increased energy demands in neurons and could activate astrocytes to increase lactate production, thereby lowering brain pH. In addition, astrocytes have been shown to play an important role in maintaining extracellular pH homeostasis by buffering increased H^+^ via neuronal activity-dependent release of bicarbonate (86). Astrocytes may also be activated by this mechanism as a compensatory mechanism for decreased extracellular pH in the specific neuropsychiatric conditions.

The results of our cell type analyses are consistent with experimental measurements showing a lower intracellular pH in astrocytes than in neurons (79, 80). Furthermore, cells of the oligodendrocyte lineage were found to have different acid-gene and alkaline-gene indices depending on the maturation stage (Figure 5C). In the cell type datasets used in these analyses, “oligodendroglia” is a mixed oligodendroglial lineage that includes mature oligodendrocytes and oligodendrocyte progenitors (89). Therefore, the immature oligodendrocyte progenitors showed a higher acid-gene index with a lower alkaline-gene index than mature oligodendrocytes, which could predict a lower intracellular pH of immature oligodendrocytes than more differentiated cells (90). Our results suggest that the expression changes of pH-associated genes are also involved in the maturation of specific cell types.

While we used pH-associated genes identified in human cortical bulk tissue to calculate acid-gene and alkaline-gene indices, the disease datasets compared included other brain and CNS regions, such as the hippocampus, striatum, cerebellum, and spinal cord. In addition, mouse datasets (CNS tissues and isolated cell types) were analyzed for human pH-associated genes. Although there is minimal variation in pH across brain regions (20, 24, 25), and mice closely mimic humans in terms of gene expression changes (78, 91), using pH-associated genes from the appropriate CNS regions, cell types, and species, if available, may increase the accuracy of predicting pH changes in each condition.

In conclusion, pH changes could be represented by specific gene expression signatures in the context of CNS disorders and different cell types in the brain. Gene expression signatures associated with pH changes may be a common endophenotype in several neuropsychiatric and neurodegenerative disorders, potentially providing new insights into the transdiagnostic molecular mechanism of these disorders.

## Data availability statement

The datasets presented in this study can be found in online repositories. The names of the repository/repositories and accession number(s) can be found in the article/Supplementary material.

## Ethics statement

Ethical review and approval was not required for the study on human participants in accordance with the local legislation and institutional requirements. Written informed consent for participation was not required for this study in accordance with the national legislation and the institutional requirements. Ethical review and approval was not required for the animal study because the publicly available gene expression datasets of humans and mice analyzed in this study were obtained from previous studies conducted elsewhere with the approval of human or animal study ethics committees.

## Author contributions

HH planned the study and performed the analysis of the datasets. TMu helped draft the manuscript. HH and TMi wrote the manuscript. TMi supervised the present study. All authors have read and approved the final manuscript.
