# Supplementary material for: The gene expression patterns as surrogate indices of pH in the brain
Source: Front Psychiatry. 2023 May 2;14:1151480. doi: 10.3389/fpsyt.2023.1151480 (PMC10185791; doi:10.3389/fpsyt.2023.1151480)
Supplement: Supplementary file 2 [file Data_Sheet_1.PDF]

The gene expression patterns as surrogate indices of pH in the brain

Hideo Hagihara[1], Tomoyuki Murano[1], and Tsuyoshi Miyakawa[1*]

[1]Division of Systems Medical Science, Center for Medical Science, Fujita Health University, Toyoake, Japan

[*]Correspondence: Tsuyoshi Miyakawa, Division of Systems Medical Science, Center for Medical Science, Fujita Health University, 1-98 Dengakugakubo, Kutsukake-cho, Toyoake, Aichi 470-1192, Japan; miyakawa@fujita-hu.ac.jp

Supplementary Figure 1

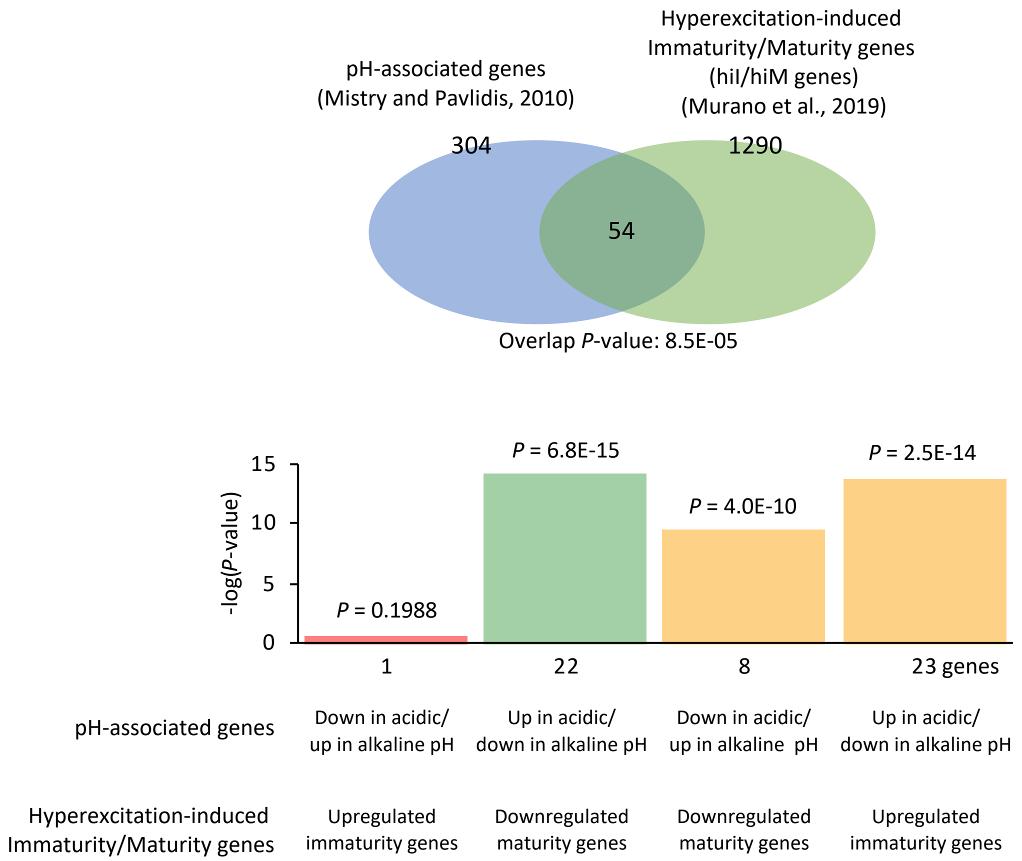

Supplementary Figure 1. Comparison between pH-associated genes and hiI/hiM genes. Venn diagram illustrates the overlap between pH-up/downregulated genes (Mistry and Pavlidis, 2010) and the hyperexcitation-induced immaturity/maturity (hiI/hiM) genes (Murano et al., 2019). Bar graph illustrates the -log of the overlap *P*-values for genes in each condition.
